# 231. Blood Neutrophil to Lymphocyte Ratio Is Associated with Mortality And Readmission in Gram Negative Bacteremia

**DOI:** 10.1093/ofid/ofad500.304

**Published:** 2023-11-27

**Authors:** Marcus Roldgaard, Thomas Benfield, Sandra Tingsgård

**Affiliations:** Copenhagen University Hospital – Amager and Hvidovre, København V, Hovedstaden, Denmark; Copenhagen University Hospital, Hvidovre, Hovedstaden, Denmark; Copenhagen University Hospital – Amager and Hvidovre, København V, Hovedstaden, Denmark

## Abstract

**Background:**

Neutrophil-Lymphocyte Ratio (NLR) in blood has been shown to be able to predict bacteremia in emergency departments and an association with mortality has been shown in patients with sepsis in intensive care units. Its potential in relation to mortality and readmission in gram negative bacteremia (GNB) is unknown.

**Methods:**

Retrospective cohort study of patients with GNB from 2018 through 2022 from four different hospitals in the greater Copenhagen area. Exclusion criteria were immunosuppression and missing NLR values on the day of blood culture. The association between NLR levels and 90-day all-cause mortality were analyzed using logistic regression models, while the association between NLR levels and 60-day readmission was analyzed using the logit link interpretation of the cumulative incidence function. Associations were expressed as odds ratios with 95% confidence intervals.

**Results:**

The study included 1763 patients. The median age was 76.8 years and 51.3% were female. The median NLR was 17.3. Urinary tract infection (UTI) was the most frequent focus and Escherichia coli was the most frequent pathogen. Significant differences in median NLR were found between age groups, pathogens, and patients with/without hypertension, liver disease, COPD, dementia, and alcohol abuse. 378 patients (21.4%) died before 90 days. 526 (29.8%) patients were readmitted to the hospital within 60 days.

The odds ratio for all-cause 90-day mortality per NLR increase by one was 1.008 (1.003-1.013, p:0.002) and for 60-day readmission, it was 1.006 (1.001-1.012, p:0.02) per NLR increase by one, corresponding to odds ratios of 1.15 (1.04-1.27, p: 0.009) and 1.11(1.01-1.23, p: 0.04) per doubling of NLR respectively. NLR was not associated with outcome when stratified for UTI and gastrointestinal infection. Discriminatory ability for mortality was limited and comparable for NLR, neutrophil and lymphocyte count, producing receiver operating characteristic curves with an area under the curve of 0.58 (0.55-0.62), 0.60 (0.55-0.62) and 0.60 (0.57-0.64).

**Figure:**
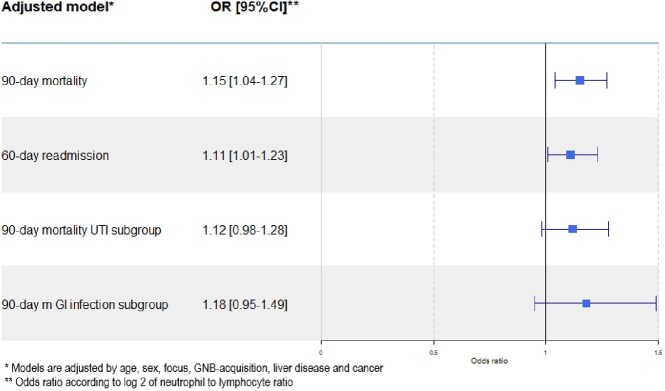

Forest plot depicting adjusted odds ratios and 95% confidence intervals for 90-day mortality and 60-day readmission

**Figure:**
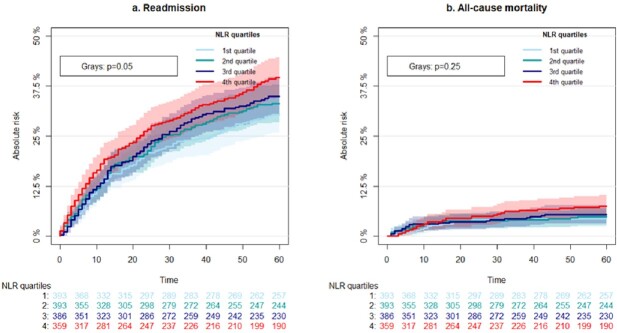

Cumulative incidence curves for 60-days readmission and mortality divided by quartiles of blood neutrophil to lymphocyte ratio

**Figure:**
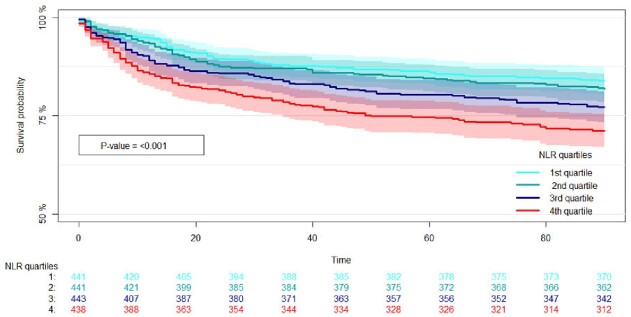

Kaplan-Meier curve of the survival probability divided by quartiles of blood neutrophil to lymphocyte ratio

**Conclusion:**

Blood neutrophil-lymphocyte ratio was associated with 90-day all-cause mortality and 60-day readmission in patients with gram negative bacteremia. However, the ratio has limited ability in predicting mortality or readmission.

**Disclosures:**

**All Authors**: No reported disclosures

